# Association Between Culture-Negative Versus Culture-Positive Sepsis and Outcomes of Patients Admitted to the Pediatric Intensive Care Unit

**DOI:** 10.7759/cureus.9981

**Published:** 2020-08-24

**Authors:** Tarek R Hazwani, Yasser M Kazzaz, Shaden Alsugheir, Shahad Aldelaijan, Futoon Alsugheir, Hamza Alali, Alaa Alsadoon, Moudi Alhamwah, Sara Alsubaiel, Bassam Alomar, Ramesh Vishwakarma

**Affiliations:** 1 Pediatric Intensive Care, Ministry of National Guard - Health Affairs, Riyadh, SAU; 2 College of Medicine, King Saud Bin Abdulaziz University for Health Sciences, Riyadh, SAU; 3 Pediatric Critical Care, Ministry of National Guard - Health Affairs, Riyadh, SAU; 4 Pediatrics, Ministry of National Guard - Health Affairs, Riyadh, SAU; 5 Emergency Medicine, Ministry of National Guard - Health Affairs, Riyadh, SAU; 6 Pediatric Infectious Diseases, Ministry of National Guard - Health Affairs, Riyadh, SAU; 7 Pediatric Emergency Medicine, Ministry of National Guard - Health Affairs, Riyadh, SAU; 8 Biostatistics, King Abdullah International Medical Research Center, Riyadh, SAU

**Keywords:** sepsis, culture, outcome, organ dysfunction, pediatric

## Abstract

Background

Sepsis remains a major cause of death, with high mortality and morbidity rates in children. The cause of mortality may be associated with several factors, including differences in cultures and the type of organism. This study was aimed at evaluating the characteristics and outcomes of negative bacterial blood culture compared to those of positive bacterial blood culture in children with severe sepsis/septic shock.

Methods

A retrospective cohort study was conducted at a pediatric intensive care unit (PICU) of a tertiary care medical center. All pediatric patients, from newborn to 14 years of age, admitted between April 2015 and January 2018 were included in the study if they fulfilled the criteria for severe sepsis/septic shock.

Results

Of the 209 patients, 30 (14.3%) had a positive bacterial blood culture whereas 179 (86.6%) had a negative bacterial blood culture. Mortality was more in positive bacterial blood culture 13 (43%) vs 35 (20%) in negative bacterial blood culture (P = 0.004). Respiratory tract infections were extremely common, present in 108 of 179 (60%) patients, and tended to result in a negative culture. The rate of organ dysfunction was higher in the positive bacterial blood culture group at admission (P = 0.01). However, the results did not reveal a significant finding related to multiorgan dysfunction syndrome (MODS) progression over three days of PICU admission (P = 0.06).

Conclusion

The negative bacterial blood culture constitutes a substantial proportion of pediatric patients with severe sepsis/septic shock. Furthermore, these pediatric patients have a lower mortality rate compared to positive bacterial blood cultures. The culture-negative sepsis group also had less organ dysfunction.

## Introduction

Severe sepsis/septic shock is a life-threatening condition, commonly managed at pediatric intensive care units (PICUs) worldwide. It is a major cause of morbidity and mortality in children, with a mortality rate of over 25% among patients in hospital admission with severe sepsis. It is estimated to affect 22 children less than 18 years of age per 100,000 person-years [1,2]. This exerts a significant impact on PICU utilization as 8% of children in PICUs meet the criteria for severe sepsis [1,3]. Most cases of mortality occur in the first 48-72 hours of PICU admission, mainly due to refractory shock and/or multiorgan dysfunction syndrome (MODS) [4,5]. Therefore, the early recognition and prediction of who are at high risk is a major contributor to sepsis morbidity and mortality and allow for timely changes in the management at PICUs, improving the overall outcomes [2].

Sepsis in children represents a spectrum of diseases involving the systemic inflammatory response syndrome in the setting of infections, escalating in septic shock to cardiovascular and organ system dysfunction in critically ill patients. Pediatric logistic organ dysfunction is a pediatric organ dysfunction score system validated for the severity classification of patients with sepsis [6,7]. The clinical course of septic shock is affected by the age, immune state of the patient, virulence of the pathogen, and hemodynamic adaptation to circulatory failure.

Although the definition and management of sepsis have changed dramatically over the last few years, the prediction of mortality of sepsis in children remains a challenge. Many markers have been studied and evaluated, such as lactate, chronic disease, cardiovascular, and respiratory derangements at PICU admission [4,8-11]. However, mortality in pediatric sepsis is mostly attributable to MODS, respiratory failure, or neurologic injury [5].

Blood culture is fundamental to the definition of sepsis and further management in children with suspected sepsis. However, blood cultures in patients with suspected sepsis are often negative, and isolation of specific organisms by culture remains challenging. Although 28%-49% of cases of severe sepsis in adults have been described to be culture-negative, data are limited on the epidemiology and outcomes in the pediatric population of those with culture-negative severe sepsis (CNSS) [12].

A limitation of larger studies on pediatric sepsis is the use of clinical definitions of sepsis, with only a few sepsis episodes being confirmed microbiologically and scant robust characterization of the presence and effect of organ dysfunction on outcomes [3,13].

Despite the publication of only a few studies that investigated the association of a positive blood culture with mortality in the pediatric population, the predictive significance of a positive blood culture in children with sepsis admitted to the PICU remains uncertain [14].

The aim of our study was to describe the patterns of culture-positive bacteria in relation to the incidence, pathogens, severity, organ dysfunctions, and outcomes to determine if a positive bacterial blood culture predicted increased morbidity and mortality among patients admitted to the PICU with severe sepsis.

Our goals were:

1- Investigate the proportion and trends of CNSS among severe sepsis patients admitted to the PICU.

2- Investigate whether or not being culture-positive is an independent predictor of mortality in patients with severe sepsis.

## Materials and methods

Design, setting, participants, and variables

We conducted a retrospective cohort study at King Abdullah Specialized Children's Hospital, a tertiary care medical center under the Ministry of National Guard - Health Affairs in Saudi Arabia, with 25 beds at the PICU involving medical and surgical cases. The present research comprises a pre-specified ancillary study of the original study: association of antibiotics administration timing with mortality in children with sepsis in a tertiary care hospital of a developing country.

Using the aforementioned database, patients aged 0 to 14 years who had been admitted between April 2015 and January 2018 were included in the study if they fulfilled the criteria for severe sepsis/septic shock. Children admitted to a separate neonatal or cardiac intensive care unit and those with advanced directives limiting resuscitation therapies at the time of sepsis recognition were excluded.

We extracted data derived from electronic medical records of pediatric sepsis patients that were collected and manually reviewed. The following variables were collected: patient demographics, Pediatric Index of Mortality 3 (PIM 3) score [15], comorbidities, hospital length of stay (LOS), PICU LOS, clinical and management characteristics (vasoactive agents and mechanical ventilation), MODS, source of infection, and culture results (blood, cerebrospinal fluid, urine, and respiratory examinations).

Data sources and definitions

We enrolled patients with severe sepsis/septic shock, as defined by the International Pediatric Sepsis Consensus Conference (IPSCC), or diagnosed by the treating physician [16]. Patients were identified using the PICU administrative database and medical records with International Classification of Diseases sepsis-related codes [17]. Organ dysfunction was defined as the IPSCC criterion [16].

CNSS was determined based on the diagnosis of severe sepsis/septic shock in the absence of positive bacterial pathogen cultures from blood. We categorized comorbidities according to the pediatric complex chronic conditions classification system, version 2 [18].

Ethical statement

This study was approved by the Institutional Review Board (IRB) of the King Abdullah International Medical Research Center (RC19/235/R). A waived consent protocol was approved by the IRB.

Interventions

Our study focused on the characteristics and outcomes of patients with CNSS compared to those with culture-positive severe sepsis (CPSS). The primary outcome measures were the proportion of patients with severe sepsis who had a negative culture, trend of CNSS patients admitted to the PICU, and incidence and trends of mortality associated with CPSS in comparison with CNSS. As for secondary outcomes, we investigated if being culture-positive is associated with more number of ventilation days and MODS incidence compared to CNSS.

Statistical analysis

All data were analyzed using SAS, version 9.3 (SAS Institute, Cary, NC), coded and de-identified, and cleaned from missing data and duplicates. Patient demographics, clinical characteristics, and outcomes are expressed as median and percentile (Q1, Q3) to describe the continuous variables of age, PIM 3 score, and progression of organ dysfunction. Frequencies and percentages were used to describe the categorical variables, such as sex, source of infection, organ dysfunction, number of comorbidities, and management (mechanical ventilation and vasopressors). PICU and hospital LOS were censored after 30 days for children still in the PICU or hospital on day 30 and for patients who died. The ventilation-free days were defined as the number of days between successful extubation and day 28; therefore, it was considered zero if the patient died before 28 days.

The chi-square test was used for correlation, and logistic regression was used to predict mortality among those who had positive and negative blood cultures. A P-value < 0.05 was considered statistically significant.

## Results

Demographics and trends of CNSS

During the study period, 206 children were admitted to the PICU with severe sepsis/septic shock. There was an almost equal sex distribution. The patients' median (Q1, Q3) ages were 19.5 (3, 73) and 11.5 (3, 63) months in the positive and negative bacterial blood culture groups, respectively. There were no statistically significant differences in the age, sex, PIM score, or source of admission between the two groups. Of the total patients, 30 (14.3%) had a positive bacterial blood culture whereas 179 (86.6%) had a negative bacterial blood culture (Table 1).

**Table 1:** Demographics ^^Chi-square test is used to calculate the P-value. ^Mann Whitney-U test is used to calculate the P-value. PIM 3: Pediatric Index of Mortality 3

Characteristic		Positive Bacterial Blood Culture (N=30)	Negative Bacterial Blood Culture (N=179)	P-value
Age (months)		11.5 (3, 63)	19.5 (3, 73)	0.52^
Neonate (less than one month)		4 (13.3)	24 (13.4)	0.3^^
Infant (1 month - Below one year)		11 (36.7)	46 (25.7)	
Children (1-14 years)		15 (50)	109 (60.9)	
Gender	Male	12 (40)	88 (49.2)	0.35^^
	Female	18 (60)	91 (50.8)	
PIM 3 score		0.0 (0.02, 0.09)	0.0 (0.01, 0.11)	0.71^
Source of admission	ER	22 (73.3)	129 (72.1)	0.89^^
	Ward	8 (26.7)	50 (27.9)	
Comorbidities	0	5 (16.7)	36 (20.1)	0.24^^
	1	17 (56.7)	69 (38.5)	
	2	5 (16.7)	34 (19.0)	
	>3	3 (10)	40 (22.3)	

Patient characteristics based on the blood culture status

With respect to comorbidities, there were no statistically significant differences between the two groups. The distribution of new-onset acute organ dysfunction varied between the two groups. Significantly fewer patients with a negative bacterial blood culture had organ dysfunction at the time of PICU admission (median, 2.9 vs. 3.0, P = 0.02). All organ dysfunctions, namely cardiac, respiratory, neurological, hematological, renal, and hepatic dysfunctions, were higher in the CPSS group. However, the results did not reveal a significant finding related to MODS progression over three days of PICU admission (median, 0.0 vs. -1.0, P = 0.06) (Tables 2, 3).

**Table 2:** Multiple organ dysfunction progression Mann Whitney-U test is used to calculate the P-value.

Characteristic	Positive Bacterial Blood Culture (N=30)	Negative Bacterial Blood Culture (N=179)	P-value
Organ dysfunction on time zero	3.0 (2, 5)	2.9 (2, 4)	0.02
Organ dysfunction during 3 days	0.0 (-1, 1)	-1.0 (-2, 0.0)	0.06

**Table 3:** Outcomes: positive bacterial blood culture vs. negative bacterial blood culture ^^Chi-square test is used to calculate the P-value. ^Mann Whitney-U test is used to calculate the P-value.

	Positive Bacterial Blood Culture	Negative Bacterial Blood Culture	P-Value
Mortality	13 (43.3)	35 (19.6)	0.004^^
Duration of mechanical ventilation, days	5.0 (1, 10)	7.0 (4, 12)	0.04^
Duration of PICU stay, days	7.0 (1, 11)	6.0 (2, 13)	0.95^
Duration of hospital stay, days	18.5 (8, 61)	15.0 (8, 37)	0.66^
Delta MODS Day 0 to 3	0.0 (-1, 1)	-1.0 (-2, 0.00)	0.06^
Ventilation free days	0.0 (0.00, 22)	18.0 (0.00, 23)	0.09^
Inotropes Days	2.5 (1, 5)	3.0 (2, 6)	0.35^

The incidence of primary bloodstream infection, and no positive culture at another body site, was found to be significantly higher in the CPSS group, seen in 16 of a total of 30 cases (P < 0.0001) and it was the most common site of infection in our study. Respiratory tract infections were extremely common, present in 108 of 179 cases (60%), and tended to result in a negative culture.

There was a statistically significant difference in the infection site between the positive and negative bacterial blood culture groups (Table 4).

**Table 4:** Infection sites based on culture status Fisher Exact test is used to calculate the P-value. CNS: central nervous system

Infection site	Positive Bacterial Blood Culture (N=30)	Negative Bacterial Blood Culture (N=179)	P-value
Primary bloodstream infection	16 (53.3)	7 (3.9)	<0.0001
Respiratory	8 (26.7)	108 (60.3)	
Skin/soft tissue	3 (10.0)	5 (2.8)	
Abdominal	1 (3.3)	17 (9.5)	
Genitourinary	1 (3.3)	10 (5.6)	
Unknown	1 (3.3)	28 (15.6)	
CNS	0	3 (1.7)	
Endocarditis	0	1 (0.6)	

Patients with a negative bacterial blood culture did not differ significantly from patients with CPSS in terms of inotropes, ventilation-free days, PICU LOS, or hospital LOS (Table 3).

Mortality based on the blood culture status

Mortality was more in CPSS 13 (43%) vs. 35 (20%) in CNSS (P = 0.004) in our study, which indicated a higher proportion of deaths in the CPSS group. The mortality status between the positive and negative bacterial blood culture groups was found to be statistically significantly different (P = 0.004) (Table 3).

## Discussion

Our study showed that only 30% of the children with severe sepsis/septic shock had a positive bacterial blood culture, which is lower than that in Morin et al.'s study [6]. The reasons for these findings may be attributed to the aggressive empiric outpatient therapy of infections and early administration of antibiotic therapy before cultures are drawn in patients when severe sepsis is recognized in the emergency room or in pediatric wards. Other potential explanations include increasing infections with viruses and fungi, particularly in immunocompromised patients, which may cause severe sepsis, as we investigated only the blood culture for bacterial organisms in our study.

In our study, patients with a negative bacterial blood culture had a significantly lower incidence of organ dysfunction at PICU admission. However, the progression of organ dysfunctions was statistically significant after three days of PICU admission, particularly cardiovascular and central nervous system failures and coagulopathy. These findings are consistent with the findings of other studies either in the pediatric or adult population [13,19], such as Lin et al.'s study, which showed that most pediatric patients with MODS developed it on day 1 (85% of all patients with MODS) and that patients with MODS on day 1 were more likely to have bacteremia [20].

To the best of our knowledge, different sites of infections result in different prognoses of sepsis progression. The incidence of primary bloodstream infection was significantly higher in the positive bacterial blood culture group, seen in 16 of a total of 30 cases. In contrast to Agyeman et al.'s study [13], our results showed that CNSS was more common than CPSS in the presence of respiratory tract infections. These findings can be attributed to most respiratory tract infections in our patients who are diagnosed with viral infections.

Inotropic support is commonly required in patients with severe sepsis/septic shock admitted to the PICU; however, limited data could be found on the association between the blood culture status and inotrope requirement in pediatric patients with severe sepsis/septic shock. Schlapbach et al. found the highest mortality in children presenting with hypotension requiring vasopressors and lactate >2 mmol/L (32.0%) [4]. However, no statistically significant difference was observed in the use of inotropes in our results between the CPSS and CNSS groups.

Our results demonstrated that the mortality rate in the positive bacterial blood culture group was higher than that in the negative bacterial blood culture group, contrary to the study by Gupta et al. [21]. Our analysis differs from Gupta et al.'s study in several aspects. Our study included only bacterial organisms in blood culture results, and we did not include the viral and fungal results. As expected, bacterial sepsis was more severe and led to more MODS, which may explain our results. Moreover, our population was limited to children aged up to 14 years. Further investigations are warranted.

This study had some limitations. First, the data collection was retrospective. Therefore, there is a risk of missing data, and information bias is not always able to establish causation. Second, we restricted the analyses to bacterial cultures only. Expansion of the analyses to include other types of organisms, i.e., viruses and fungi, could be useful for understanding the association between the culture status and outcome. Third, this was a single-center study; therefore, we have to consider the differences in national health care systems and population demographics for its generalizability. Finally, using mortality as an outcome does not capture the impact of culture results on patients surviving with major long-term morbidity.

## Conclusions

Our findings indicate that a negative bacterial blood culture constitutes a substantial proportion of pediatric patients with severe sepsis/septic shock. Furthermore, these pediatric patients have a lower mortality rate compared to positive bacterial blood cultures. The culture-negative sepsis group also had less organ dysfunction. A better understanding of bacterial sepsis in pediatrics and the reasons for worse associated outcomes of CPSS should be the focus of future studies.
